# Magnetic resonance imaging of RRx-001 pharmacodynamics in preclinical tumors

**DOI:** 10.18632/oncotarget.18455

**Published:** 2017-06-12

**Authors:** Natarajan Raghunand, Jan Scicinski, Gerald P. Guntle, Bhumasamudram Jagadish, Eugene A. Mash, Elizabeth Bruckheimer, Bryan Oronsky, Ronald L. Korn

**Affiliations:** ^1^ Moffitt Cancer Center, Tampa, Florida, USA; ^2^ EpicentRx, Inc., San Diego, California, USA; ^3^ Arizona Cancer Center, The University of Arizona, Tucson, Arizona, USA; ^4^ Department of Chemistry and Biochemistry, The University of Arizona, Tucson, Arizona, USA; ^5^ Imaging Endpoints LLC, Scottsdale, Arizona, USA

**Keywords:** RRx-001, gadolinium, MRI, redox, BOLD

## Abstract

RRx-001 is an anticancer agent that subjects cancer cells to reactive oxygen/nitrogen species (ROS/RNS) and acts as an epigenetic modifier. We have used a thiol-bearing MRI contrast agent, Gd-LC7-SH, to investigate the pharmacodynamics of RRx-001 in CHP-100 Ewing's Sarcoma, HT-29 colorectal carcinoma, and PANC-1 pancreatic carcinoma xenografts in SCID mice. Binding of Gd-LC7-SH to the Cys^34^ residue on plasma albumin prolongs retention in the tumor microenvironment and increases tumor enhancement on MRI. Mice were imaged by MRI and *in vivo* T1 maps acquired 50 min (T1_50 min_) after injection of 0.05 mmol/kg Gd-LC7-SH (i.v.) at baseline and 1, 24, and 72 h post-treatment with 10 mg/kg RRx-001 (i.v.). Consistent with an indirect thiol-modifying activity of RRx-001, tumor T1_50 min_ at 1 h post-drug was significantly longer than pre-drug tumor T1_50 min_ in all three tumor models, with the T1_50 min_ remaining significantly longer than baseline through 72 h post-drug in the HT-29 and PANC-1 tumors. The T1_50 min_ of CHP-100 tumors recovered to baseline by 24 h post-drug, suggesting a robust anti-oxidant response to the RRx-001 challenge that was presaged by a marked increase in perfusion at 1 h post-drug measured by DCE-MRI. MRI enhanced with Gd-LC7-SH provides a mechanistically rational biomarker of RRx-001 pharmacodynamics.

## INTRODUCTION

RRx-001 is a member of the novel dinitroazetidine-containing class of anticancer agents that perturbs the thiol redox potential of the cancer cell, subjecting it to damaging ROS/RNS [1] and acting as an epigenetic modifier [2, 3]. With the successful completion of a first-in-human Phase 1 study [4], RRx-001 is currently being investigated in a number of Phase 2 clinical trials as an epigenetic primer to resensitize or “episensitize” patients to previously effective but now refractory therapies [5–10].

**Figure 1:**
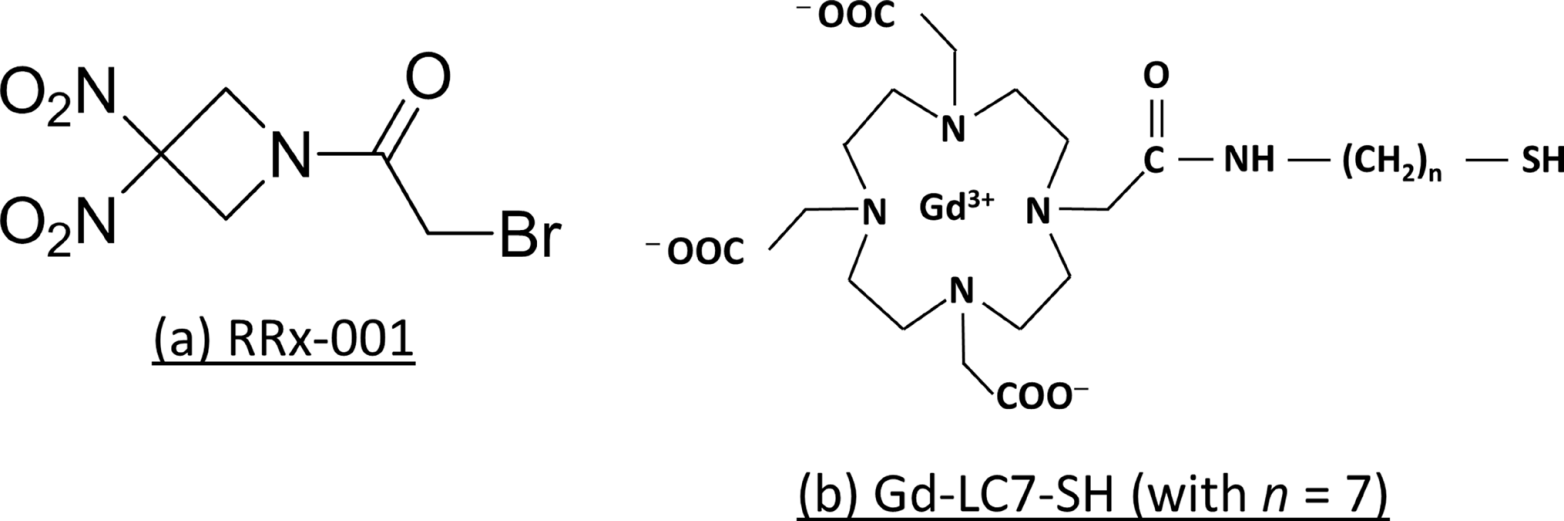
Structures of RRx-001 and Gd-LC7-SH

Pre-clinical studies have shown that RRx-001 (Figure 1A) selectively, rapidly, and irreversibly binds glutathione and a specific thiol on hemoglobin, resulting in a hypoxia-mediated, NO-releasing pro-oxidant effect in red blood cells that is transferred to tumors while sparing normal tissue [11–14]. Increases in intratumoral oxidative stress mediated by RRx-001 have previously been measured *in vitro* with the fluorescent probe 2′,7′-dichlorofluorescein diacetate, and by studying the activation of the Nrf2-ARE antioxidant signaling pathways in tumor cells, specifically the nuclear translocation of Nrf2 and the expression of its downstream enzymes HO-1 and NQO1 by RRx-001 [12]. In addition, Nrf2-ARE antioxidant signaling pathway activation was demonstrated *in vivo* by molecular imaging of tumor cells co-expressing pARE-Firefly luciferase and pCMV-Renilla luciferase-mRFP [13]. Treatment of C3H mice bearing SCC VII tumors with RRx-001 alone, or in combination with radiation, produced significant dose- and time-dependent increases in tumor perfusion and blood volume measured by microbubble-enhanced ultrasound imaging. The growth delay in these tumors produced by RRx-001 treatment could be abrogated by co-administration of N-acetylcysteine [12]. Pre-clinical and clinical data indicate that RRx-001 metabolism leads to the depletion of reduced glutathione and generation of nitric oxide. Downstream sequelae of RRx-001 reactivity include the oxidative modification of catalytic cysteine thiol groups and inactivation of crucial enzymes in the tumor microenvironment. Studying the tumor redox properties of RRx-001 will provide insight into the selection of biomarkers to identify patient populations that may benefit from the drug and to identify modalities that will enable pharmacodynamic monitoring of response in the clinic [15].

**Figure 2:**
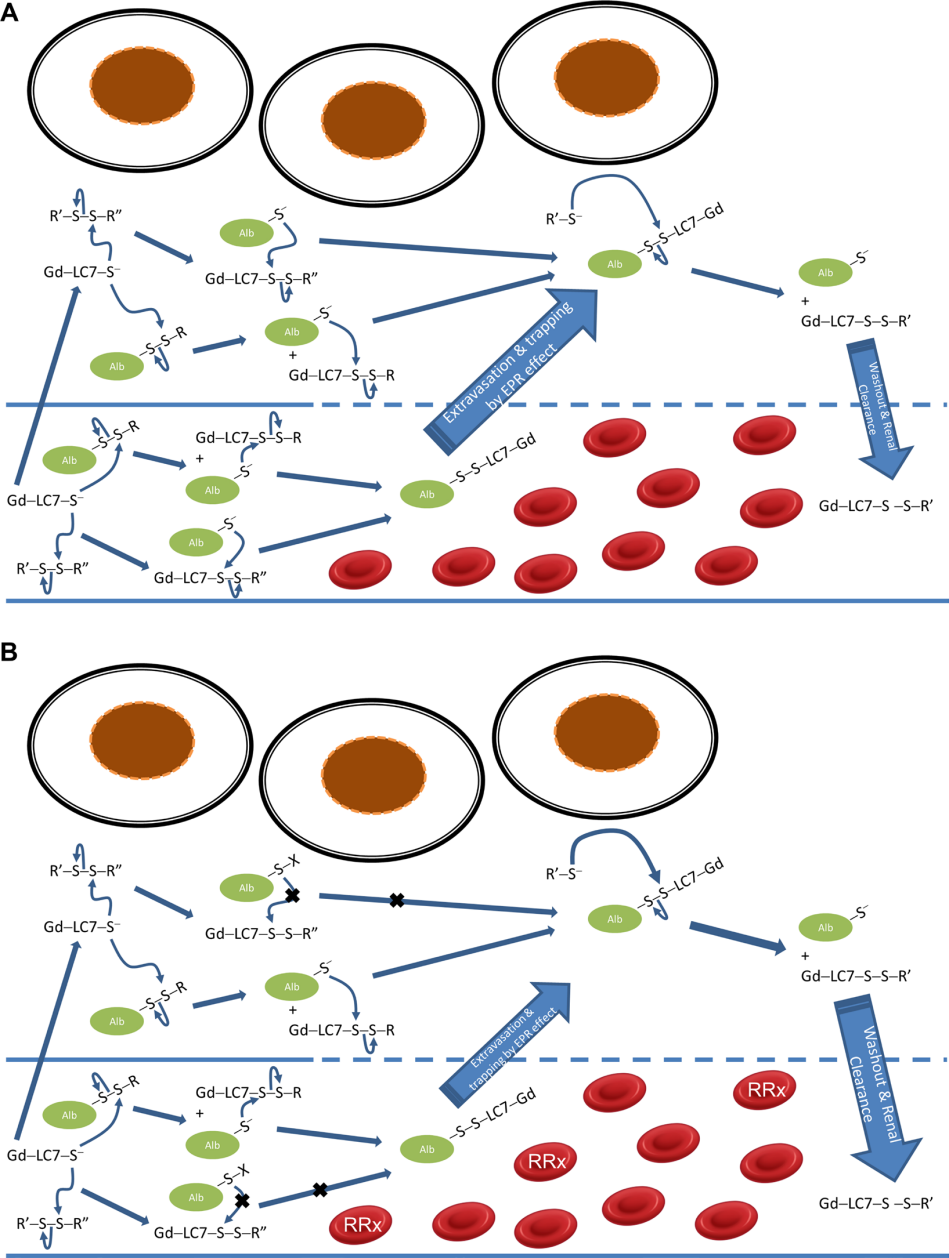
Mechanism of RRx-001 drug effect on decreasing retention of Gd-LC7-SH in the tumor microenvironment The pKa of the thiol group in the conserved Cys^34^ residue on albumin is ≈ 5, such that at physiological pH about two-thirds of it exists as thiolate anion (Alb-S^-^) while the rest is disulfide-bonded to small molecule thiols (Alb-S-S-R) [17]. For the purposes of clarity we have depicted small molecule thiols also as thiolate anions; actual reactions may involve both the thiolate and thiol forms, depending on the pK_a_ and their relative nucleophilicity. (**A**) Two possible reactions by which i.v. administered Gd-LC7-SH spontaneously binds to plasma albumin at Cys^34^ are depicted. Late signal enhancement on MRI is primarily due to albumin-bound gadolinium trapped in the tumor interstitium by the Enhanced Permeability & Retention (EPR) effect. Bound gadolinium can be released from the albumin by competing small molecule thiols R’-SH. (**B**) RRx-001 binds to the Cys^93^ residue on hemoglobin in a subset of RBCs (indicated by ‘RRx’). Localization of RRx-001 bound RBCs to the tumor produces oxidative stress with release of ROS, RNS and free iron, which in turn lead to oxidative and nitrosative modifications of albumin-Cys^34^ (indicated by –S–X where X = NO, OH, O_2_H or O_3_H). This decreases binding of Gd-LC7-SH to albumin-Cys^34^ and retention in the tumor interstitium. EPR retention of albumin-bound Gd-LC7-SH may be further decreased if there is compensatory production of small molecule thiols R’-SH by oxidatively stressed tumor cells, which can competitively release Gd-LC7-SH from the albumin resulting in accelerated washout from the tumor microenvironment.

In this study we have interrogated the glutathione- and cysteine-depleting effects of RRx-001 using MRI enhanced with Gd-LC7-SH (Figure 1B), a chelate of gadolinium that exhibits increased longitudinal MRI relaxivity (r1 relaxivity) upon binding with human serum albumin (HSA). The binding of Gd-LC7-SH to HSA is inhibited by homocysteine in a manner consistent with single site binding, and 2D NMR spectroscopy of the binding of a congener molecule Gd-LC6-SH (*n* = 6 in Figure 1B) reveals that site to be the conserved Cys^34^ residue of HSA [16]. Albumin is known to combine with cysteine and homocysteine in plasma to form the respective mixed disulfides at its Cys^34^ residue. In a careful study of the binding of homocysteine to albumin, Jacobsen and colleagues provide evidence that when reduced homocysteine enters circulation, it attacks albumin-Cys^34^-S-S-Cys to first form albumin-Cys^34^ thiolate anion, which then reacts with homocysteine-cysteine mixed disulfide to form albumin-bound homocysteine [17]. Based on their findings, in Figure 2A we propose mechanisms by which i.v. administered Gd-LC7-SH may bind to plasma albumin and subsequently be retained in the tumor interstitium to produce prolonged MRI image enhancement. Albumin-Cys^34^ is expected to be the major macromolecular site for the binding of Gd-LC7-SH since it is known to be the largest extracellular depot for carrying small molecule thiols *in vivo* [17, and references therein]. It should however be noted that the mechanism proposed in Figure 2 will also hold for the binding of Gd-LC7-SH to any other macromolecular thiol or immobile site such as exofacial protein thiols. In Figure 2B we hypothesize a mechanism by which RRx-001 treatment will abrogate such retention of Gd-LC7-SH in the tumor microenvironment.

In a previous work we carried out a detailed study of the MRI tracer kinetics of Gd-LC6-SH and compared it against Gd-DTPA-BMA (a small molecule) and Galbumin^®^ (a macromolecule) [18]. In that study we demonstrated that delayed (50–60 min post-injection) enhancement on MRI is insensitive to injected Gd-LC6-SH dose provided the injected dose is 0.05 mmole Gd/Kg or higher (a “saturating” dose). The albumin-binding properties and MRI relaxivities of Gd-LC6-SH and Gd-LC7-SH are similar [16], and therefore in the current study we have measured tumor T1 changes 50 min following administration of 0.05 mmole/Kg Gd-LC7-SH. At this delayed time point any free Gd-LC7-SH or its low molecular weight disulfide forms will have been largely cleared from circulation and a pseudo-steady-state between free and macromolecule-bound Gd-LC7-SH expected to exist in the tumor microenvironment [18]. We have measured T1_50 min_, the longitudinal MRI relaxation time of tumor 50 min post-administration of 0.05 mmol/kg Gd-LC7-SH, before and after treatment of tumor-bearing mice with RRx-001. We hypothesize that the redox modifying activity of RRx-001 can be monitored through changes in tumor T1_50 min_ on Gd-LC7-SH-enhanced MRI. In three tumor models the tumor T1_50 min_ post-drug was longer than pre-drug tumor T1_50 min_, indicative of decreased retention of Gd-LC7-SH post-treatment in the tumor. Additionally, post-drug T1_50 min_ of CHP-100 tumors recovered to baseline values significantly faster than in PANC-1 and HT-29 tumors; this recovery was associated with an acute increase in CHP-100 tumor perfusion measured by Dynamic Contrast-Enhanced (DCE-) MRI at 1 h post-drug. We also investigated Blood Oxygen Level Dependent (BOLD) MRI as a potential biomarker of the NO-related vascular effects of RRx-001.

## RESULTS

### Gd-LC7-SH MRI results

**Figure 3:**
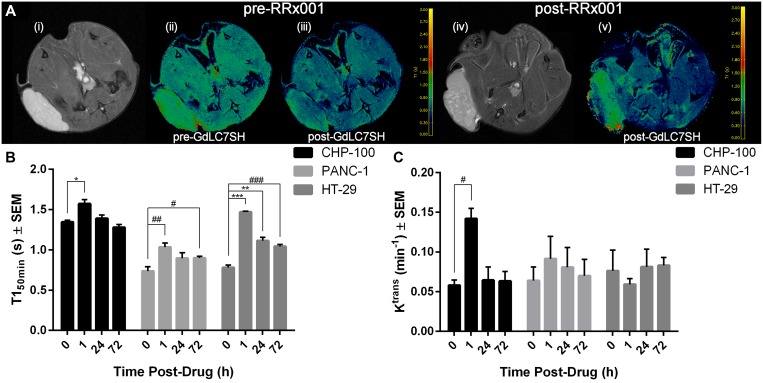
Gd-LC7-SH MRI of 3 tumor models (**A**) Anatomic reference images and example T1 maps before and 50 min after Gd-LC7-SH of a mouse bearing a HT-29 tumor xenograft; the color scale runs from 0 s (dark blue) through 3 s (yellow). Before treatment with RRx-001 (panel (iii)) the T1 of both tumor and muscle are significantly lower post-Gd-LC7-SH relative to pre-Gd-LC7-SH (panel (ii)). 24 h after RRx-001 exposure the T1_50 min_ of tumor is higher than expected (panel (v)), consistent with a rapid clearance of Gd-LC7-SH from the treated tumor. (**B**) Tumor T1 (mean ± S.E.M., *n* = 4) measured 50 min post-Gd-LC7-SH, before *vs.* 1–72 h after treatment with RRx-001 (10 mg/kg, *i.v.*). In all three tumors, the T1_50 min_ at 1 h post-treatment with RRx-001 was significantly higher than pre-treatment T1_50 min_ (^*^p = 0.014, ^##^*p* = 0.006, ^***^p = 7 × 10^−5^). In HT-29 and PANC-1 tumors this effect of RRx-001 on tumor T1_50 min_ was apparent even at 72 h post-drug (^#^*p* = 0.047, ^**^p = 0.001, ^###^*p* = 7 × 10^−4^). (**C**) K^trans^, a measure of tumor perfusion measured by DCE-MRI, before *vs.* after treatment with RRx-001 (10 mg/kg, *i.v.*). In CHP-100 tumors, tumor perfusion was significantly higher 1 h post-treatment with RRx-001 relative to baseline (^#^*p* = 0.003).

As depicted in Figure 2A, the fraction of gadolinium that is bound to albumin (or other macromolecular target) in the tumor will experience delayed washout due to the EPR effect. This prolonged retention of Gd-LC7-SH is apparent on tumor T1 maps measured by MRI before *vs.* 50 min after administration of Gd-LC7-SH to the animal (Figure 3A, panels (ii) *vs.* (iii)). As hypothesized in Figure 2B, blocking of the binding of Gd-LC7-SH to albumin by RRx-001 treatment will result in faster washout of Gd-LC7-SH. This faster washout of Gd-LC7-SH is apparent as a longer post-drug tumor T1_50 min_ relative to pre-drug tumor T1_50 min_ (Figure 3A, panels (v) *vs.* (iii)). As summarized in Figure 3B, the T1_50 min_ at 1 h post-treatment with RRx-001 was significantly longer than pre-treatment T1_50 min_ in CHP-100 tumors (1.57 ± 0.05 s *vs.* 1.35 ± 0.02 s, *p* = 0.014), PANC-1 tumors (1.04 ± 0.05 s *vs.* 0.74 ± 0.05 s, *p* = 0.006) and HT-29 tumors (1.47 ± 0.01 s *vs.* 0.78 ± 0.03 s, *p* = 7 × 10^−5^). Tumor T1_50 min_ remained significantly longer than pre-treatment T1_50 min_ even 72 h post-drug in PANC-1 tumors (0.90 ± 0.02 s *vs.* 0.74 ± 0.05 s, *p* = 0.047) and HT-29 tumors (1.04 ± 0.02 s *vs.* 0.78 ± 0.03 s, *p* = 7 × 10^−4^), pointing to a prolonged oxidative effect of RRx-001 on the microenvironment in these tumors (Figure 3B). The T1_50 min_ in CHP-100 tumors was statistically indistinguishable from pre-treatment T1_50 min_ at 24 h post-drug and had recovered to baseline levels by 72 h post-drug, suggesting a robust anti-oxidant response to the drug in these tumors (Figure 3B).

### DCE-MRI results

DCE-MRI data were collected for only the first 5 min post-second-bolus of Gd-LC7-SH, and the K^trans^ parameter calculated from fitting this data to the Tofts model is therefore weighted primarily toward perfusion rather than microvascular permeability [19]. Pre-treatment K^trans^ was similar in all three tumor models (Figure 3C). In the CHP-100 tumors, K^trans^ was markedly higher 1 h post-treatment relative to the pre-treatment value (Figure 3C, 0.14 ± 0.01 min^−1^ *vs.* 0.06 ± 0.01 min^−1^, *p* = 0.003), indicating an acute increase of tumor perfusion in response to RRx-001 which was not apparent at later times post-drug. No significant change in tumor K^trans^ relative to baseline was observed in the PANC-1 and HT-29 tumors at any times post-drug (Figure 3C). Tumor v_e_ and v_p_ were not significantly altered, relative to baseline, by RRx-001 treatment in any of the three tumor models at any times post-drug (data not shown).

### BOLD MRI results

**Figure 4:**
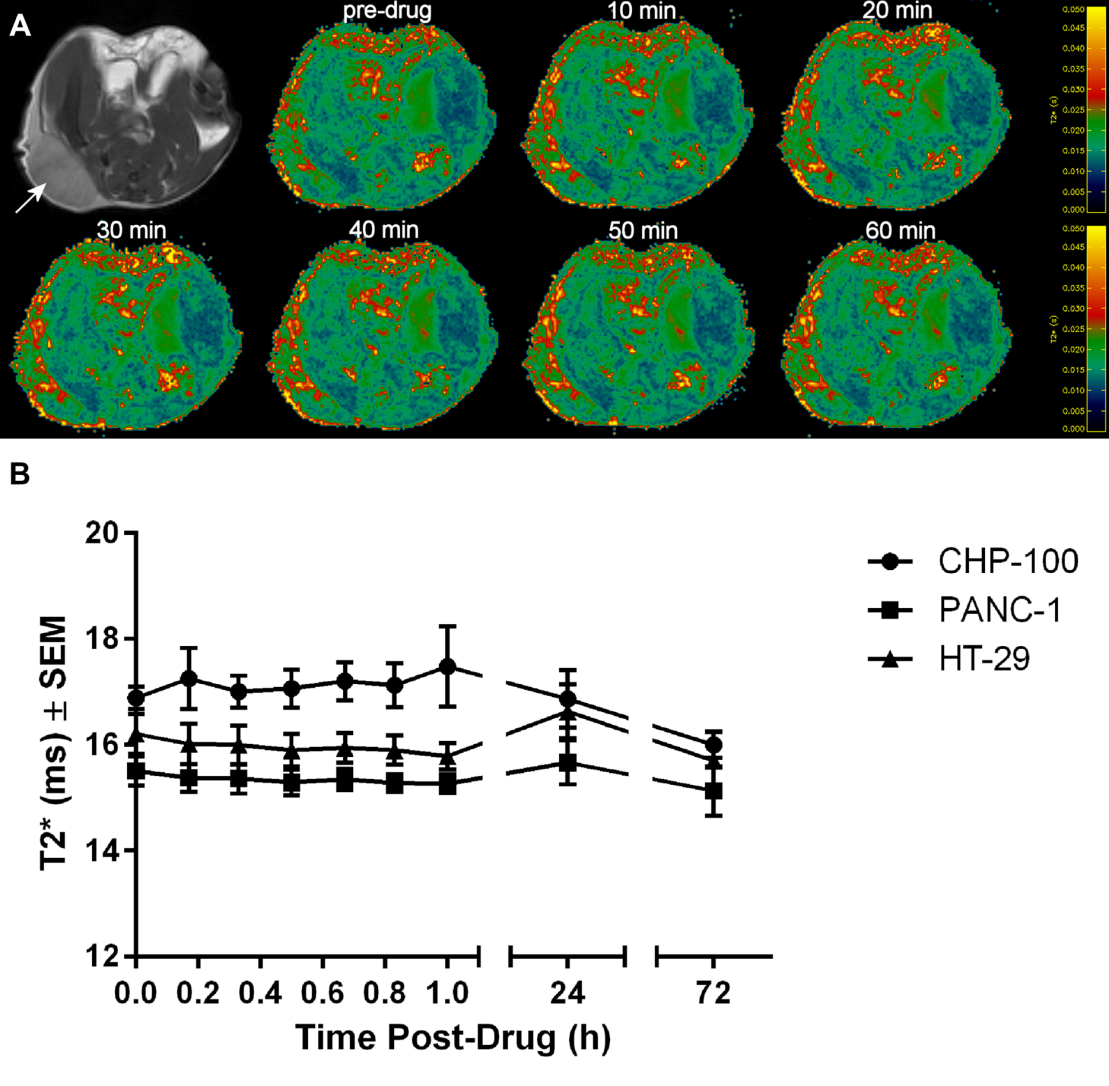
BOLD MRI of 3 tumor models (**A**) Anatomic reference images and example T2* maps acquired in a mouse bearing a CHP-100 tumor (arrow); the color scale runs from 0 ms (dark blue) through 50 ms (yellow). (**B**) T2* maps were acquired before and through 60 min after RRx-001 treatment, and again at 24 h and 72 h post-drug. The variation of tumor T2* (mean ± S.E.M., *n* = 4) before *vs.* after treatment with RRx-001 (10 mg/kg, *i.v.*).

The transverse relaxation time T2* measured by MRI is sensitive to changes in deoxyhemoglobin content arising from either local vasodilation or local changes in oxygen tension [20]. RRx-001 is known to alkylate a thiol on hemoglobin and trigger downstream effects on erythrocytes and NO production [11]. Therefore, tumor T2* was measured before, during, and after RRx-001 administration (Figure 4A). No significant acute changes in T2* relative to baseline were observed in any of the tumor models between 0–60 min following RRx-001 (Figure 4B). Tumor T2* was also measured 24 h and 72 h post-drug, though any apparent T2* changes at these two time points relative to baseline are difficult to interpret, given that field inhomogeneity contributions to T2* would have been different on Day 1 (0–60 min post-drug), Day 2 (24 h post-drug), and Day 4 (72 h post-drug). Subject to that caveat, the T2* of CHP-100 tumors was significantly lower at 72 h post-drug relative to baseline (Figure 4B, 15.99 ± 0.17 ms *vs.* 16.89 ± 0.16 ms, *p* < 0.0001). Subject to the same caveat regarding differing contributions of field inhomogeneity to T2* measured during different MRI sessions, the T2* of CHP-100 tumors was significantly longer than those of PANC-1 and HT-29 tumors in the 0–60 min post-drug window (Figure 4B). This is potentially reflective of better tumor oxygenation (lower tumor deoxyhemoglobin) in CHP-100 tumors at baseline and post-drug.

## DISCUSSION

Although the structure of RRx-001 may suggest that this agent binds to intratumoral thiols by covalent and irreversible alkylation leading to direct changes in the tumor redox environment, the metabolism of RRx-001 [11], characterized by a very short half-life in whole blood and by irreversible and selective binding with glutathione and the beta-Cys-93 residue on hemoglobin, suggests a more complex mechanism of action. Binding of RRx-001 to hemoglobin and glutathione depletion lead to an increase in the oxidative stress of red blood cells exposed to RRx-001. While the RRx-001-GSH adduct is excreted rapidly (terminal half-life ~30 min), the RRx-001-bound hemoglobin adduct persists for the lifetime of the red blood cell [11–14]. Oxidatively and nitrosatively stressed erythrocytes are characterized by changes in hemoglobin oxygen affinity arising from hemoglobin conformational changes, highly potentiated nitrite reductase activity under hypoxic conditions [21–22], and, with very high oxidation levels, membrane scrambling manifested by externalization of phosphatidyl serine. These changes lead to aggregation of the RRx-001-bound RBCs, followed by internalization by tumor vasculature [23], leading to a release of heme, iron, and oxidized lipid products, that is, ROS and RNS, as well as changes in tumor blood flow.

In a previous study of congeners of Gd-LC7-SH, we found that the binding of Gd-LC3-SH (*n* = 3 in Figure 1B) through Gd-LC7-SH (*n* = 7 in Figure 1B) to HSA is consistent with single-site binding at the conserved Cys^34^ residue of HSA, and that the homocysteine-inhibitable binding affinity for HSA increases in the order Gd-LC3-SH through Gd-LC7-SH [16]. For this reason we used Gd-LC7-SH in the present study to investigate the oxidative effects of RRx-001 on tumor tissue *in vivo*. In all three tumor models, RRx-001 treatment resulted in longer tumor T1_50 min_ values at 1 h post-drug relative to pre-drug values, which is consistent with accelerated washout of Gd-LC7-SH due to drug effect, as hypothesized in Figure 2. This effect was apparent even at 72 h post-drug in the two carcinoma models, PANC-1 and HT-29. In the CHP-100 Ewing sarcoma model, however, the tumor T1_50 min_ at 24 h and 72 h post-drug was not significantly different from pre-drug values. This suggests that this tumor mounts a strong anti-oxidant response to the RRx-001 challenge, possibly stemming from the known activation of NRF2 signaling in sarcomas [24–25]. One component of an anti-oxidant response would be improved perfusion, and there was a marked increase in the K^trans^ parameter calculated from DCE-MRI of CHP-100 Ewing sarcoma xenografts at 1 h post-drug treatment. DCE-MRI data were collected to 5 min post-injection of a second bolus of Gd-LC7-SH, and contrast agent distribution kinetics in the early phase are thought to be dominated by perfusion rather than by microvascular permeability and leakage [19]. In contrast to the acute response observed in CHP-100 tumors, no significant change in perfusion was measured at any time points post-drug in the PANC-1 and HT-29 carcinoma xenografts. Unlike in carcinomas, the vascular network in Ewing sarcomas is formed by a combination of angiogenesis and vasculogenesis. While the development of a vascular net by angiogenesis is common with many tumor types, vasculogenesis, the process by which bone marrow-derived cells are recruited into newly developing vasculature, leads to a more mature vascular phenotype [26], potentially as a result of enhanced PDGFr signaling [27]. This more mature vasculature, together with reduced thrombospondin [28] levels that modulate the physiological effects of nitric oxide through the inhibition of cGMP production [29], may result in a phenotype that is acutely responsive to the presence of nitric oxide, potentially explaining the acute changes in perfusion induced by RRx-001 in the CHP-100 tumors.

T2* of CHP-100 tumors measured in the 0–60 min post-drug time period were significantly longer than the T2* of PANC-1 and HT-29 tumors. While several factors can impact T2*, longer T2* values may reflect lower levels of deoxyhemoglobin in CHP-100 tumors relative to PANC-1 and HT-29 tumors, in turn suggesting that these tumors are better perfused. We have previously investigated changes in tumor blood flow induced by RRx-001 using contrast-enhanced ultrasound [12] and immunohistochemistry (IHC) of pimonidazole [30]. Pimonidazole staining showed that, while parts of the tumor experienced an almost total shutdown of blood flow, the overall level of hypoxia within tumors was not significantly altered by RRx-001 treatment, suggesting a redistributive effect rather than outright vascular disruption [12]. This may explain why we did not see an acute change in average tumor T2* in response to RRx-001 in any of the three tumor models.

In conclusion, MRI enhanced with Gd-LC7-SH provides a non-invasive pharmacodynamic marker of the redox modulatory activity of RRx-001 in three pre-clinical tumor models. DCE-MRI provides evidence of an acute increase of tumor perfusion in CHP-100 Ewing sarcoma tumors following RRx-001 therapy. We are exploring the addition of DCE-MRI as an investigational pharmacodynamic biomarker to ongoing clinical studies of RRx-001 (NCI Clinical Trials Identifier NCT02215512, [31]), while continuing with the development of Gd-LC7-SH. Clinically, localized soft-tissue sarcomas (STS) are commonly treated with preoperative radiotherapy, and hypoxia in STS has been reported to be associated with radioresistance [32]. Our results in a pre-clinical model of Ewing sarcoma, if borne out in a wider study, offer hope that RRx-001 may be useful as a radiosensitizer for neoadjuvant radiotherapy of STS.

## MATERIALS AND METHODS

### Mice, tumors, and drug

All animal experiments were conducted in accordance with the guidelines of the Institutional Animal Care and Use Committee (IACUC) at the University of Arizona. CHP-100 Ewing's Sarcoma, HT-29 colorectal carcinoma, and PANC-1 pancreatic carcinoma cells were originally obtained from ATCC, and xenografts on flanks of 6-week old female Severe Combined Immunodeficient (SCID) mice were established by subcutaneous injection of 1 × 10^7^ cells. 18 mice were inoculated with each tumor cell line, of which the 12 mice with the most similar mean tumor volumes were stratified into 3 equal treatment groups when the tumors were approximately 250–400 mm^3^: Gd-LC7-SH MRI group 1 (*n* = 4), Gd-LC7-SH MRI group 2 (*n* = 4), and BOLD MRI group 3 (*n* = 4). Animals were monitored every three days for general health, body weight, and tumor volume by caliper measurement. RRx-001 was obtained from EpicentRx, Inc., [33] and prepared as a 5 mg/mL solution in saline containing dimethylacetamide (7.5% v/v) and polyethylene glycol 400 (15% v/v). RRx-001 solution was used within six hours of preparation and administered to mice at a dose of 10 mg/kg (0.05 mL i.v. to a 25 g mouse) as described below. Gd-LC7-SH was synthesized as described previously [16], prepared as a 25 mM solution in injectable saline at pH 7.4, and administered to mice at a dose of 0.05 mmole/kg (0.05 mL i.v. to a 25 g mouse) as described below.

### Magnetic resonance imaging

Mice were imaged on a 7 Tesla Bruker Biospec^®^ small animal MRI scanner. Mice were anesthetized using isoflurane (1.5–2.5% in O_2_, 1 L/min) and cannulated at the tail vein with an i.v. line of ~25 μL dead volume for administration of Gd-LC7-SH or RRx-001. A pressure transducer pad for monitoring respiration was placed under the anesthetized animal, and the mouse was gently secured in a plastic holder. The animal was loaded into a 25-mm-ID small animal imaging Litz coil (Doty Scientific, Columbia, SC) and the entire assembly was centered inside the MRI magnet. Animal body temperature was maintained using a forced warm air system and monitored during imaging using a fluoroptic rectal temperature probe (SA Instruments, Stony Brook, NY). Isoflurane was adjusted manually to maintain animal respiration rate largely in the 35–45 breaths/min range during scanning. Scout images were acquired for planning axial slices to be imaged through the tumor, followed by acquisition of T2-weighted anatomic reference images in the final slice geometry. For computing longitudinal relaxation time (T1) maps, eight gradient-echo (GRE) images were acquired using the following parameters at a scan time of 80 seconds per image: repetition time (TR) = 78 milliseconds, echo time (TE) = 3.5 milliseconds, matrix = 256 × 256, field of view (FOV) = 3 × 3 cm, slice thickness = 2 mm, number of averages (NA) = 4, and flip angles (FA) = 5°, 15°, 25°, … 75° (8 flip angles). DCE-MRI was performed by GRE imaging with the following parameters: TR = 78 milliseconds, TE = 3.5 milliseconds, FA = 75°, NA = 1, number of repetitions = 15, scan time = 5 min. BOLD MRI was performed by acquiring multi-echo gradient-echo (mGRE) images with the following parameters: TR = 300 ms, TE = 4, 12, 20, … 60 ms (8 echoes), FA = 25°, NA = 8, matrix = 128 × 128, FOV = 3 × 3 cm, slice thickness = 2 mm.

### Gd-LC7-SH MRI, group 1

**Figure 5:**
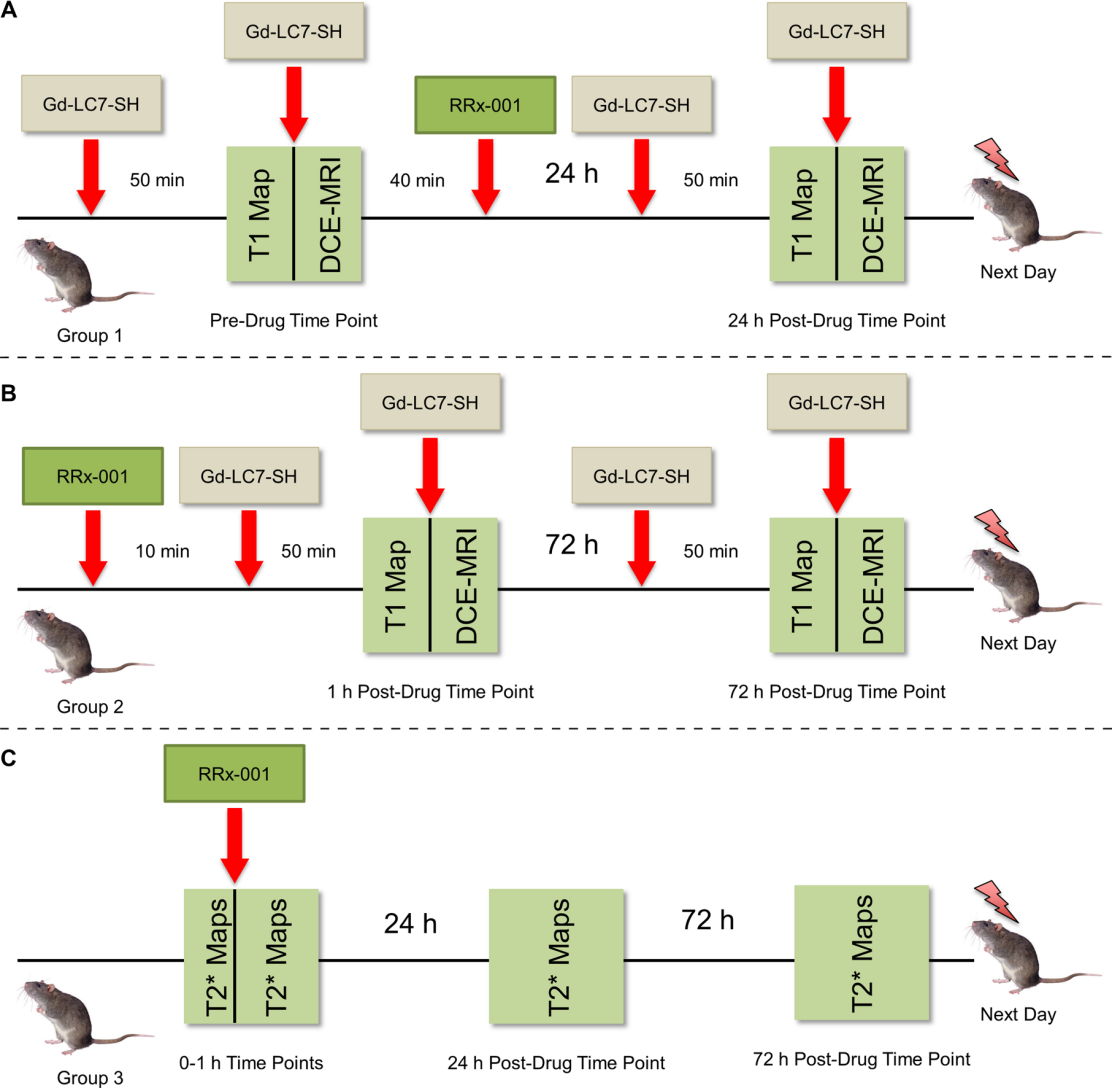
Designs of the Gd-LC7-SH MRI, DCE-MRI, and BOLD MRI experiments

Mice in this group were imaged before treatment (0 h) and 24 h post-treatment as in Figure 5A. Mice were prepared for MRI as described above and T1 maps of the tumor were acquired 50 min post-injection of 0.05 mmol/kg Gd-LC7-SH (i.v.) in untreated mice. At this point a second bolus of 0.05 mmol/kg Gd-LC7-SH with 0.1 mL saline flush was administered and DCE-MRI was performed. At the end of scanning, the mouse was kept anesthetized outside the magnet for approximately 40 min, with the *i.v.* line still in place, at which time RRx-001 was injected. This delay was to allow the previously injected Gd-LC7-SH to either form mixed disulfides with targets *in vivo* or be cleared from the animal, thereby avoiding direct reaction between RRx-001 and Gd-LC7-SH. Mice in this group were re-imaged 24 h post-administration of RRx-001, after which they were sacrificed by cervical dislocation under anesthesia (4% isoflurane) and the tumors harvested.

### Gd-LC7-SH MRI, group 2

Mice in this group were imaged 1 h and 72 h post-treatment, as in Figure 5B. Mice were prepared for MRI as described above and administered RRx-001, followed by a 0.1 mL saline flush. The purpose of the saline flush was to clear the *i.v.* line of residual RRx-001 and to prevent direct reaction with Gd-LC7-SH that was administered 10 min later (0.05 mmol/kg). Unreacted RRx-001 has a very short half-life in blood, on the order of seconds [11, 34], and so the 10 min delay was considered sufficient to prevent direct reaction between RRx-001 and Gd-LC7-SH *in vivo*. Mice were then transferred into the magnet, and T1 maps of the tumor were acquired 50 min post-injection of Gd-LC7-SH (1 h post-drug). At this point, a second bolus of 0.05 mmol/kg Gd-LC7-SH with a 0.1 mL saline flush was administered and DCE-MRI was performed. Mice in this group were re-imaged 72 h post-administration of RRx-001, after which they were sacrificed by cervical dislocation under anesthesia (4% isoflurane) and the tumors harvested.

### BOLD MRI, group 3

Mice in this group were imaged before, during, and through to 60 min after treatment, 24 h post-treatment, and 72 h post-treatment, as in Figure 5C. Mice were prepared for MRI as described above, and two mGRE image sets were acquired before treatment. Then RRx-001 was administered in-magnet via the *i.v.* line, and additional mGRE datasets were acquired through to 1 h post-drug. Mice in this group were re-imaged at 24 h and 72 h post-administration of RRx-001, after which they were sacrificed by cervical dislocation under anesthesia (4% isoflurane) and the tumors harvested.

### MRI data analysis

The signal intensities in the 8 GRE images acquired with 8 flip angles were fitted by non-linear regression on a pixel-by-pixel basis to the gradient-echo signal equation to compute T1 maps. T2* maps were calculated by linear regression of the natural logarithm of the pixel-by-pixel signal intensities in the mGRE images *vs.* TE. DCE-MRI was performed following a second bolus of Gd-LC7-SH administered ≈ 60 min after the first bolus. We have previously reported a method for quantitative analysis of dual-bolus DCE-MRI images in which the residual time-varying signal intensity from the first bolus was estimated by fitting a biexponential washout function and subtracted from the time-varying signal intensity following the second bolus [35]. Based on the *in vivo* behavior of a related molecule, Gd-LC6-SH [18, 36], we have assumed that at ≈ 60 min post-injection of Gd-LC7-SH the signal enhancement from the first bolus is essentially time-invariant during the 5 min DCE-MRI measurement following the second bolus. DCE-MRI signal intensities were therefore corrected by simply subtracting the pre-second-bolus signal intensity, rather than a time-varying washout function, from each time point. Pixel-by-pixel pharmacokinetic analysis of this corrected DCE-MRI data was performed using a modified Tofts model [19]. Three physiologically relevant model parameters were fitted for each pixel, the volume transfer constant (K^trans^), volume fraction of the extravascular extracellular space (v_e_), and plasma volume fraction (v_p_) [37].
